# Divergent Attitudes Toward COVID-19 Vaccine vs Influenza Vaccine

**DOI:** 10.1001/jamanetworkopen.2023.49881

**Published:** 2023-12-21

**Authors:** Gillian K. SteelFisher, Mary G. Findling, Hannah L. Caporello, Ericka McGowan, Laura Espino, Jazmyne Sutton

**Affiliations:** 1 Department of Health Policy and Management, Harvard T. H. Chan School of Public Health, Boston, Massachusetts; 2 Association of State and Territorial Health Officials, Arlington, Virginia; 3 National Public Health Information Coalition, Canton, Georgia; 4 SSRS, Glen Mills, Pennsylvania

## Abstract

This survey study examines attitudes toward COVID-19 and influenza vaccines among US adults.

## Introduction

Although the emergency phase of the COVID-19 pandemic is over, it leaves the legacy of more complex and potentially deadly respiratory virus seasons going forward.^1,2^ As the viral landscape shifts, there is new urgency to understand US adults’ views on relevant vaccines, including whether they perceive annual vaccines similarly, or whether there are differences that may impact coadministration and communications. To guide programmatic and policy decision-making, we examined attitudes toward COVID-19 and influenza vaccines in this survey study.

## Methods

Data come from a survey conducted July 7 to 16, 2023, among a nationally representative, probability-based sample of US adults aged 18 years or older. It was designed by researchers at Harvard T. H. Chan School of Public Health (HSPH) with input from Association of State and Territorial Health Officials and National Public Health Information Coalition staff and was determined to be exempt by the institutional review board of HSPH. Participants provided consent through the survey. We followed American Association for Public Opinion Research (AAPOR) reporting guidelines. eAppendix 1 and eAppendix 2 in Supplement 1 contain methodological details.

Respondents reported attitudes toward COVID-19 and influenza vaccine effectiveness and safety, vaccination intentions, and major reasons for hesitancy. We examined data for adults overall and adults aged 50 years and older, who may have higher risk of severe illness. Data were weighted to US Census parameters to produce nationally representative estimates (eTable in Supplement 1). Differences were examined in Stata statistical software version 15.0 (StataCorp) using 2-tailed *t *tests. Statistical significance was set at *P* < .05.

## Results

Among 3232 invited participants, 1430 (44%) completed the survey. Nearly equal shares said that COVID-19 vaccines (42%) and influenza vaccines (40%) are very effective at protecting against serious illness or hospitalization (all percentages are weighted) (Table 1). Views diverged on vaccine safety, where a higher share said influenza vaccines are very safe compared with COVID-19 vaccines (55% vs 41%). Similarly, intentions differed: 49% said they are very likely to get an influenza vaccine this season, compared with 36% saying the same for an updated COVID-19 vaccine. Patterns were similar among adults aged 50 years and older.

**Table 1.** Attitudes Toward COVID-19 and Influenza Vaccines Among a Nationally Representative Sample of US Adults^a^

Attitude	Participants, weighted %			
	All adults aged ≥18 y (n = 1430)		Only adults aged ≥50 y (n = 659)	
	COVID-19 vaccine	Influenza vaccine	COVID-19 vaccine	Influenza vaccine
Effectiveness^b^				
Very effective	42	40	50	49
Somewhat effective	28	38^c^	25	35^c^
Not too effective	13^c^	7	12^c^	5
Not at all effective	7^c^	4	6	4
Safety^d^				
Very safe	41	55^c^	51	65^c^
Somewhat safe	29	29	24	23
Not too safe	9^c^	4	9^c^	4
Not at all safe	9^c^	4	8^c^	2
Vaccination intentions^e^				
Very likely	36	49^c^	49	62^c^
Somewhat likely	18	15	15^c^	9
Not too likely	17^c^	13	13^c^	8
Not at all likely	28^c^	22	23	21

^a^ Data come from a July 2023 nationally representative, probability-based online and telephone survey of 1430 US adults aged 18 years and older. Weighted percentages are displayed and results may not add up to 100% because of rounding and because responses of “Don’t know” and “Refused” are included in the total but are not displayed. See eAppendix 1 and eAppendix 2 in Supplement 1 for methodological details.

^b^ The full question was, “In general, how effective are [COVID-19 vaccines/flu vaccines] for most adults in protecting the person getting vaccinated from getting seriously ill or having to be hospitalized with [COVID-19/the flu]?”

^c^ Value is significantly higher than comparison group (COVID-19 or influenza) at *P* < .05.

^d^ The full question was, “In general, how safe are [COVID-19 vaccines/flu vaccines] for most adults?”

^e^ The full question was, “Now imagine that, in the future, an updated COVID-19 vaccine or booster becomes available each year, and public health officials recommend all adults get it each fall. In this scenario, how likely would you be to get an updated COVID-19 vaccine this coming fall, in 2023? How likely are you to get a flu vaccine this coming flu season, which is expected to run from this October in 2023 to next April, in 2024?”

Among more hesitant adults (those not very likely to get vaccinated), concerns about updated COVID-19 and influenza vaccines differed (Table 2). Thematic differences were evident, with greater shares of COVID-19 vaccine–hesitant adults citing major reasons for hesitancy as wanting more research done, worries about vaccine safety and effectiveness, and believing they are already well-protected through prior vaccination or infection, compared with influenza vaccine–hesitant adults. In addition, despite the same government agencies and pharmaceutical companies promoting COVID-19 and influenza vaccines, higher shares of COVID-19 vaccine–hesitant adults cited distrust of these agencies and companies. Results were similar among adults aged 50 years and older. Subanalyses by degrees of hesitancy (somewhat, not too, and not at all likely) show that many differences in reasons for hesitation held along the continuum, with greater proportions having concerns about COVID-19 vaccines at each degree of hesitancy (data not shown).

**Table 2.** Major Reasons for Vaccine Hesitancy Among Those Not Very Likely to Get COVID-19 or Influenza Vaccines in the Future^a^

Reason for vaccine hesitancy	Participants, weighted %			
	All adults aged ≥18 y		Only adults aged ≥50 y	
	COVID-19 vaccine (n = 437-777)	Influenza vaccine (n = 535-579)	COVID-19 vaccine (n = 180-295)	Influenza vaccine (n = 173-199)
You would want to see more research done on the [updated COVID-19/flu] vaccine	60	24	62	25
You would be worried about the safety of [updated COVID-19 vaccines/the flu vaccine]	51	25	56	29
You do not trust the government agencies that promote [updated COVID-19 vaccines/the flu vaccine]	45	27	55	33
You do not think [an updated COVID-19 vaccine/a flu vaccine] would be very effective [in protecting against COVID-19]/the flu	40	27	39	28
You would rather get natural immunity from getting [COVID-19/the flu]	38	37	43	35
You do not trust the companies that make [updated COVID-19 vaccines/the flu vaccine]	38	25	39	25
You feel like people are expected to get too many vaccines in general	35	30	41	31
You already had [COVID-19/the flu] and you think that will be enough protection	27	13	23	12
You think the previous [COVID-19 vaccines or boosters/flu vaccines] you have already gotten will be enough protection^b^	26	13	24	11
You do not think you would be likely to get very sick if you got [COVID-19/the flu]	25	27	24	24
You think getting [another COVID-19/another flu] vaccine would be too much for your immune system^b^	22	15	22	15
You had a bad reaction to a previous [COVID-19/flu] vaccine [or booster]^b^	19	18	19	19
You would not have time to get it or schedule around possible side effects like a fever	14	16	10	10

^a^ Data come from a July 2023 nationally representative online and telephone survey of 1430 US adults. Weighted percentages are displayed according to the percentage of adults aged 18 years and older expressing major reasons for COVID-19 hesitancy. Adults who reported weaker vaccination intentions (ie, somewhat, not too, or not at all likely to get a COVID-19 vaccine this fall or an influenza vaccine this influenza season) were given a list of 10 potential reasons to choose from (each participant was asked a random subset of 10 reasons among a list of 14 total reasons). Sample sizes are shown in ranges because of variation in the number of respondents asked each reason, due to randomization.

^b^ COVID-19 questions were asked for only participants who had received at least 1 dose of a COVID-19 vaccine last year.

## Discussion

The COVID-19 pandemic was the largest scale infectious disease outbreak in a generation, killing more than 1 million US residents in just 3 years. By historical standards, a vaccine was developed in record time, with a high safety profile and high effectiveness—greater than that of the influenza vaccine in many years.^3^ Despite this, US adults, including older adults most at risk of serious illness, are more hesitant about COVID-19 vaccines than influenza vaccines, with divergent concerns, as shown in this survey study.

Thus, health professionals should expect limited demand for COVID-19 vaccines and moderate interest in influenza vaccines. Where coadministration is offered, communications should lead with the more popular influenza vaccine, provide consistent messaging on safety and effectiveness of both vaccines, and address vaccine-specific beliefs, such as the limits of protection from prior COVID-19 infection. Public health agencies should also work with trusted messengers to build trust.^4^

This study’s limitations include the use of cross-sectional, self-reported data, and the potential for nonresponse bias. Vaccine hesitancy in the US is not monolithic, and it is essential that health professionals and communicators address nuances in public opinion to promote vaccine uptake this season and beyond.

## Notes

Supplement 1. **eAppendix 1.** Questionnaire Language and Analysis Details **eAppendix 2.** Sampling, Weighting, and Margin of Sampling Error Details **eTable.** Comparison Between Sample and National Benchmarks for Key Demographics

Supplement 2.
Data Sharing Statement
