# Supplementary material for: Divergent Attitudes Toward COVID-19 Vaccine vs Influenza Vaccine
Source: JAMA Netw Open. 2023 Dec 21;6(12):e2349881. doi: 10.1001/jamanetworkopen.2023.49881 (PMC10739094; doi:10.1001/jamanetworkopen.2023.49881)
Supplement: Supplement 1. — eAppendix 1. Questionnaire Language and Analysis Details eAppendix 2. Sampling, Weighting, and Margin of Sampling Error Details eTable. Comparison Between Sample and National Benchmarks for Key Demographics [file jamanetwopen-e2349881-s001.pdf]

Supplemental Online Content

Steelfisher GK, Findling MG, Caporello HL, McGowan E, Espino L, Sutton J. Divergent attitudes toward COVID-19 vaccine vs influenza vaccine. *JAMA Netw Open*. 2023;6(12):e2349881. doi:10.1001/jamanetworkopen.2023.49881

eAppendix 1. Questionnaire Language and Analysis Details

eAppendix 2. Sampling, Weighting, and Margin of Sampling Error Details

eTable. Comparison Between Sample and National Benchmarks for Key Demographics

This supplemental material has been provided by the authors to give readers additional information about their work.

© 2023 Steelfisher GK et al. *JAMA Network Open.*

eAppendix 1: Questionnaire Language and Analysis Details

Details on Questionnaire Development

The questionnaire was developed using American Association of Public Opinion Research best practices for survey research.[1] The content and wording of questions, response options, and question order and flow were all developed after reviewing prior surveys.[2] Before fielding, the questionnaire was reviewed for bias, balance, and comprehension, with pretesting among a subsample of participants via telephone interviews to improve clarity.

Details on the Analytic Approach

Analyses in this paper focused on respondents at the top end of scales (for example, comparing those "very likely" to get vaccinated versus those not "very likely" to get vaccinated), because using top-end response categories better predicts behaviors—including vaccination—than other response combinations (such as when "very likely" and "somewhat likely" to get vaccinated are combined).[3] Differences in reasons for hesitancy between COVID-19 and flu vaccines persisted whether we defined vaccine hesitant as "somewhat likely", "not too likely", and "not at all likely," or defined it only as those "not too likely" and "not at all likely".

Questions

Questionnaire Language	Response Categories
**Attitudes**	
In general, how effective are COVID-19 vaccines for most adults in protecting the person getting vaccinated from getting seriously ill or having to be hospitalized with COVID-19?	Very effective<br>Somewhat effective<br>Not too effective<br>Not effective at all
In general, how effective are flu vaccines for most adults in protecting the person getting vaccinated from getting seriously ill or having to be hospitalized with the flu?	Very effective<br>Somewhat effective<br>Not too effective<br>Not effective at all
In general, how safe are COVID-19 vaccines for most adults?	Very safe<br>Somewhat safe<br>Not too safe<br>Not at all safe

[1] American Association for Public Opinion Research (AAPOR). Best Practices for Survey Research [Internet].; 2022. https://www.aapor.org/Standards-Ethics/Best-Practices.aspx#best3

[2] STAT-Harris Poll, Weighted to the U.S. General Adult Population - Propensity. Fielded December 17-19, 2021. http://freepdfhosting.com/a3a5f4a2d4.pdf; Annenberg Public Policy Center of the University of Pennsylvania. Annenberg Science Knowledge (ASK) Survey, July 12-July 18, 2022, N=1,580 Adults.; 2022. https://cdn.annenbergpublicpolicycenter.org/wp-content/uploads/2022/08/W7b_Appendix_D6_Aug22.pdf

[3] See: SteelFisher et al, *Health Affairs,* 2023, doi: /10.1377/hlthaff.2022.01204; Moucheraud, Guo, Macinko, *Health Affairs*, 2021,40(8):1215–1224; Dennison, *Political Studies Review*. 2019;17(4):436-446; Visser, Krosnick, Norris, Chapter 8 in *Political Psychology*. 2017:217-259; SteelFisher et al., *Am J Obstet Gynecol,* 2011;204(6)S:S116-S123.

© 2023 Steelfisher GK et al. *JAMA Network Open.*

In general, how safe are flu vaccines for most adults?	Very safe<br>Somewhat safe<br>Not too safe<br>Not at all safe
**Vaccination intentions**<br>Now imagine that, in the future, an updated COVID-19 vaccine or booster becomes available each year, and public health officials recommend all adults get it each fall. In this scenario, how likely would you be to get an updated COVID-19 vaccine this coming fall, in 2023?	Very likely<br>Somewhat likely<br>Not too likely<br>Not at all likely
How likely are you to get a flu vaccine this coming flu season, which is expected to run from this October in 2023 to next April, in 2024?	Very likely<br>Somewhat likely<br>Not too likely<br>Not at all likely
**Reasons for vaccine hesitancy (asked among those only "somewhat likely," "not too likely," or "not at all likely" to get an updated COVID-19 or flu vaccine)**	
There are lots of reasons people may not be [if "somewhat" or "not too" likely: very] likely to get an updated COVID-19 vaccine this coming fall. Are each of the following a major reason, a minor reason, or not a reason at all that you would not be [if "somewhat" or "not too" likely: very] likely to get an updated COVID-19 vaccine this coming fall?<br><br>a. I would want to see more research done on the updated COVID-19 vaccine<br>b. I think getting another COVID-19 vaccine would be too much for my immune system<br>c. I would rather get natural immunity from getting COVID-19<br>d. I think the previous COVID-19 vaccines or boosters I have already gotten will be enough protection<br>e. I already had COVID-19 and I think that will be enough protection<br>f. I wouldn't have time to get it or schedule around possible side effects like a fever<br>g. I would be worried about the safety of updated COVID-19 vaccines<br>h. I don't think an updated COVID-19 vaccine would be very effective in protecting against COVID-19<br>i. I don't think I would be likely to get very sick if I got COVID-19<br>j. I had a bad reaction to a previous COVID-19 vaccine or booster<br>k. I don't trust the companies that make updated COVID-19 vaccines<br>l. I don't trust the government agencies that promote updated COVID-19 vaccines<br>m. I feel like people are expected to get too many vaccines in general	Major reason<br>Minor reason<br>Not a reason
There are lots of reasons people may not be [if "somewhat" or "not too" likely: very] likely to get a flu vaccine this coming flu season. Are each of the following a major reason, a minor reason, or not a reason at all that you are not [if "somewhat" or "not too" likely: very] likely to get a flu vaccine this coming flu season?<br>a. I would want to see more research done on the flu vaccine<br>b. I think getting another flu vaccine would be too much for my immune system<br>c. I would rather get natural immunity from getting the flu<br>d. I think the previous flu vaccines I have already gotten will be enough protection<br>e. I already had the flu and I think that will be enough protection<br>f. I wouldn't have time to get it or schedule around possible side effects like a fever	Major reason<br>Minor reason<br>Not a reason

© 2023 Steelfisher GK et al. *JAMA Network Open.*

g.	I would be worried about the safety of the flu vaccine
h.	I don't think the flu vaccine would be very effective
i.	I don't think I would be likely to get very sick if I got the flu
j.	I had a bad reaction to a previous flu vaccine
k.	I don't trust the companies that make the flu vaccine
l.	I don't trust the government agencies that promote the flu vaccine
m.	I feel like people are expected to get too many vaccines in general

© 2023 Steelfisher GK et al. *JAMA Network Open.*

eAppendix 2. Sampling, Weighting, and Margin of Sampling Error Details

Respondents for the poll were reached through the SSRS Opinion Panel,[4] a nationally representative probability-based web panel. Given that this is a probability-based panel, findings are statistically projectable to the general U.S. adult population. A total of 1,328 surveys were completed by panelists online and 102 interviews were completed with panelists who do not have access to the internet, or who do not use it. The sample was weighted in stages. The first stage of the weighting was the application of a base weight to account for different selection probabilities and response rates across sample strata. In the second stage sample demographics were post-stratified to match population parameters. Comparisons between the survey sample and national benchmarks for key demographics are shown in the next section. The design effect for this survey was 1.61, which means the margin of error for the entire sample is ±3.3 percentage points at the 95% confidence interval.

The participation rate for this survey was expected due to the rapid response nature of fielding the survey, and prior research suggests that the resulting data are comparable to longer-term, higher-response surveys when reweighted to key population parameters.[5] After weighting, there was less than a 1-percentage-point difference between the survey sample and national sources for all demographic characteristics (see below).

[4] https://ssrs.com/opinion-panel/

[5] See, for example, Kohut et al. Assessing the Representativeness of Public Opinion Surveys. Pew Research Center. 2012. http://www.people-press.org/2012/05/15/assessing-the-representativeness-of-public-opini on-surveys; Mercer A, Lau A. Comparing Two Types of Online Survey Samples. Pew Research Center; 2023. https://www.pewresearch.org/methods/2023/09/07/comparing-two-types-of-online-survey-samples/; Keeter S, Hatley N, Kennedy C, Lau A. What Low Response Rates Mean for Telephone Surveys. Pew Research Center; 2017. https://www.pewresearch.org/wp-content/uploads/2017/05/RDD-Non-response-Full-Report.pdf.

© 2023 Steelfisher GK et al. *JAMA Network Open.*

eTable. Comparison Between Sample and National Benchmarks for Key Demographics

The eTable below shows key demographics of the sample compared to benchmark data.

- Gender, age, education, race/ethnicity, and region benchmarks were derived from 2022 Current Population Survey (CPS)[6].
- The civic engagement benchmark was derived from September 2019 Volunteering and Civic Life Supplement data[7] from the Current Population Survey (CPS).
- The population density benchmark came from Census Planning Database 2021[8].
- The internet frequency benchmark is from the National Public Opinion Reference Survey (NPORS) for Pew Research Center - May 23 to Sept 6, 2022[9].

Weights were trimmed to prevent individual interviews from having too much influence on final results.

As shown in the eTable below, differences between the weighted sample and benchmarks all fell within 1 percentage point.

eTable. Comparison Between Sample and National Benchmarks for Key Demographics

Variable		Benchmark %	Weighted %
Gender	Male	48.6%	48.4%
	Female	51.4%	51.6%
Age (in years)	18-24	11.4%	11.4%
	25-34	17.5%	17.7%
	35-44	16.9%	17.3%
	45-64	32.3%	32.7%
	65+	22.0%	20.9%
Education	Less than HS	9.6%	9.5%
	HS diploma	29.2%	29.3%
	Some College	26.4%	26.8%
	College+	34.8%	34.4%
Region	North East	17.4%	17.9%
	Midwest	20.6%	20.8%
	South	38.3%	38.1%
	West	23.7%	23.3%
Civic Engagement	Engaged	32.7%	33.1%
	Not engaged	67.3%	66.9%
Race/Ethnicity	White, Non-Hispanic	62.0%	62.1%
	Black, Non-Hispanic	12.0%	11.8%
	Hispanic, Native Born	8.3%	8.5%
	Hispanic, Foreign Born	8.9%	8.7%
	Asian, non-Hispanic	6.3%	6.3%
	Other, non-Hispanic	2.5%	2.5%
Density Quintiles	1	20.0%	19.4%
	2	20.0%	20.2%
	3	20.0%	20.5%
	4	20.0%	20.0%
	5	20.0%	19.9%

[6] Sarah Flood, Miriam King, Renae Rodgers, Steven Ruggles, J. Robert Warren and Michael Westberry. Integrated Public Use Microdata Series, Current Population Survey: Version 10.0 [dataset]. Minneapolis, MN: IPUMS, 2022. https://doi.org/10.18128/D030.V10.0
[7] Civically engaged respondents are defined as those who have volunteered in the past 12 months or who talk to their neighbors daily. https://www.census.gov/programs-surveys/cps/about/supplemental-surveys.html
[8] https://www.census.gov/topics/research/guidance/planning-databases/2021.html
[9] https://www.pewresearch.org/methods/fact-sheet/national-public-opinion-reference-survey-npors/

© 2023 Steelfisher GK et al. *JAMA Network Open.*
