# Supplementary material for: Divergent Attitudes Toward COVID-19 Vaccine vs Influenza Vaccine
Source: JAMA Netw Open. 2023 Dec 21;6(12):e2349881. doi: 10.1001/jamanetworkopen.2023.49881 (PMC10739094; doi:10.1001/jamanetworkopen.2023.49881)
Supplement: Supplement 2. — Data Sharing Statement [file jamanetwopen-e2349881-s002.pdf]

Data Sharing Statement

Steelfisher. Divergent Attitudes Toward COVID-19 Vaccine vs Influenza Vaccine. *JAMA Netw Open*. Published December 21, 2023. doi:10.1001/jamanetworkopen.2023.49881

Data
Data available: No

Additional Information
Explanation for why data not available: Additional publications from these data are forthcoming.
